# Intracellular pH regulation: characterization and functional investigation of H^+^ transporters in *Stylophora pistillata*

**DOI:** 10.1186/s12860-021-00353-x

**Published:** 2021-03-08

**Authors:** Laura Capasso, Philippe Ganot, Víctor Planas-Bielsa, Sylvie Tambutté, Didier Zoccola

**Affiliations:** 1 Centre Scientifique de Monaco, 8 quai Antoine 1er, 98000 Monaco, Monaco; 2 Sorbonne Université, Collège Doctoral, F-75005 Paris, France

**Keywords:** H^+^ transport, Reef-building corals, pH regulation, Gene expression, Ocean acidification

## Abstract

**Background:**

Reef-building corals regularly experience changes in intra- and extracellular H^+^ concentrations ([H^+^]) due to physiological and environmental processes. Stringent control of [H^+^] is required to maintain the homeostatic acid-base balance in coral cells and is achieved through the regulation of intracellular pH (pH_i_). This task is especially challenging for reef-building corals that share an endosymbiotic relationship with photosynthetic dinoflagellates (family Symbiodinaceae), which significantly affect the pH_i_ of coral cells. Despite their importance, the pH regulatory proteins involved in the homeostatic acid-base balance have been scarcely investigated in corals. Here, we report in the coral *Stylophora pistillata* a full characterization of the genomic structure, domain topology and phylogeny of three major H^+^ transporter families that are known to play a role in the intracellular pH regulation of animal cells; we investigated their tissue-specific expression patterns and assessed the effect of seawater acidification on their expression levels.

**Results:**

We identified members of the Na^+^/H^+^ exchanger (SLC9), vacuolar-type electrogenic H^+^-ATP hydrolase (V-ATPase) and voltage-gated proton channel (H_v_CN) families in the genome and transcriptome of *S. pistillata*. In addition, we identified a novel member of the H_v_CN gene family in the cnidarian subclass Hexacorallia that has not been previously described in any species. We also identified key residues that contribute to H^+^ transporter substrate specificity, protein function and regulation. Last, we demonstrated that some of these proteins have different tissue expression patterns, and most are unaffected by exposure to seawater acidification.

**Conclusions:**

In this study, we provide the first characterization of H^+^ transporters that might contribute to the homeostatic acid-base balance in coral cells. This work will enrich the knowledge of the basic aspects of coral biology and has important implications for our understanding of how corals regulate their intracellular environment.

**Supplementary Information:**

The online version contains supplementary material available at 10.1186/s12860-021-00353-x.

## Background

Coral reefs are among the most valuable ecosystems on earth, harbouring more than one-third of ocean biodiversity and providing economic benefits to tropical coastal nations worldwide [1]. Scleractinian corals are the major constructors of coral reefs, and most of them have mutualistic relationships with endosymbiotic dinoflagellate (family Symbiodinaceae)-symbiotic corals that provide photosynthetic products that support coral metabolism, growth and reproduction [2–4]. Despite their environmental significance, scleractinian corals face many challenges to their survival, including ocean acidification (OA), as a result of rising carbon dioxide levels in the atmosphere [5]. The effects of OA on the coral calcification rate and skeletal growth, including varied species-specific responses, have been previously documented [6–9]. Most of these effects have been proposed to be linked to acid-base regulatory processes and to lead to altered ionic concentration and energy expenditure and allocation [10]. Although acid-base regulatory processes might modulate physiological responses to OA, the pH regulatory proteins responsible for acid-base homeostasis are still poorly characterized in corals.

Corals are diploblastic animals, which means that they are made of two cell layers, an ectoderm and an endoderm, separated by a layer of mesoglea. Both ectoderm and endoderm are present in the oral and aboral tissue located on either side of the gastrovascular cavity (coelenteron) [11]. Each tissue possesses several cell subtypes that achieve acid-base homeostasis via pH regulation within physiological boundaries compatible with cell functioning [12]. This is especially challenging for symbiotic corals, as photosynthesis significantly increases the pH of coral cells [13, 14]. Symbiotic corals can experience large variations in extracellular pH (pH_e_) due to physiological (e.g., respiration, calcification and photosynthesis) and environmental (e.g., metabolism of reef-associated organisms, tides, water flow, upwelling and ocean acidification) factors. For example, symbiont photosynthesis and respiration of both the host and symbiont drive wide pH_e_ variations (from pH 8.5 to 6) in the internal fluid of the coelenteron [13–18], and exposure to acidified seawater decreases the pH_e_ in the extracellular calcifying medium (ECM) [9], where calcification occurs. These pH_e_ variations can also have an impact on the regulation of intracellular pH (pH_i_). For example, decreases in the pH_e_ of the ECM (from 8.3 to 7.8) under acidified seawater conditions affect the pH_i_ of calicoblastic cells. Despite these variations, coral cells are able to maintain their pH_i_ within narrow limits (7.1–7.4) [9], showing that corals possess efficient pH_i_ regulatory mechanisms that account for this stability.

Under pH_i_ stress, an appropriate response depends on the ability to sense acid-base disturbances and react through acid-base transport mechanisms [19]. Over the previous years, a number of acid-base cellular sensors, e.g., the acid-base sensing enzyme soluble adenylyl cyclase (sAC), and transport proteins have been proposed in corals on the basis of those characterized in vertebrates [20–22]. Acid-base transport proteins, which control pH_i_ regulation, fall into two groups, namely, HCO_3_^−^ (solute carrier-SLC transporter family 4 and 26) and H^+^ membrane transporters [23]. Based on the energy source used for H^+^ extrusion, H^+^ membrane transporters can be further divided into transporters, pumps and channels. Transporters can move H^+^ against their electrochemical gradient by coupling H^+^ transport with pre-existing ion gradients as an energy source. This group includes the SLC9 family, also known as Na^+^/H^+^ exchangers, which harness the electrochemical gradient of Na^+^ maintained by Na^+^/K^+^-ATPase [24]. The SLC9 family can be further divided into three subfamilies: subfamily A 1–9 (cation proton antiporter 1, CPA1), subfamily B 1–2 (cation proton antiporter 2, CPA2) and subfamily C 1–2 (Na-transporting carboxylic acid decarboxylase, NaT-DC) [25]. The second group of H^+^ membrane transporters in charge of pH_i_ regulation includes H^+^ pumps that allow H^+^ to move against its concentration gradient by coupling H^+^ transport to ATP hydrolysis. Vacuolar-type electrogenic H^+^-ATP hydrolases (V-ATPases), which transport H^+^ via V_0_ V-ATPase subunit-a [26], and plasma membrane Ca^+ 2^-ATPase (PMCA), which extrudes Ca^+ 2^ in exchange for H^+^ [19, 27, 28], belong to this group. Although some studies claim that pH_i_ regulation is not the primary role of these H^+^ pumps in most mammalian cells [19], others have suggested the opposite [29–32]. Members of the last group, the H^+^ channels, allow H^+^ to passively diffuse down its electrochemical gradient whenever the regulatory gate is open and includes voltage-gated proton channels (H_v_CN) [33, 34].

Despite their fundamental importance in pH_i_ regulation, the investigation of these H^+^ transporters is incomplete and includes only a limited number of marine species. For example, SLC9 and V-ATPase proteins have been identified in the transcriptome of calcifying primary mesenchymal cells of the sea urchin [35] and in the gills of fishes, where they are believed to play a role in acid-base regulation of the branchial epithelium [36, 37]. In addition, in white shrimp (*Litopenaeus vannamei*), squid (*Sepioteuthis lessoniana*), and sea anemone (*Anemonia viridis*), SLC9 has been shown to play a role in the response to low-pH stress [38–40]. In corals, among the acid-base transport proteins in charge of pH_i_ regulation, only HCO_3_^−^ transporters have been characterized [20], whereas information regarding H^+^ membrane transporters is limited to Ca^+ 2^-ATPase [41] and V_1_ V-ATPase subunit B [42].

In the present study, we provide a full characterization of the genomic structure, domain topology and phylogeny of the principal H^+^ transporters that might be involved in the intracellular pH regulation and maintenance of cellular physiological homeostasis in the symbiotic coral *Stylophora pistillata*. These transporters include Na^+^/H^+^ exchangers, which are ion transporters that concurrently transport Na^+^ into the cell and H^+^ out of the cell; voltage-gated H^+^ channels (H_v_CN), which represent a specific subset of proton channels that have voltage- and time-dependent gating and thus only open to extrude H^+^ from the cell; and V_0_ V-ATPase subunit-a, which connects the two portions (V_0_ and V_1_) of the multi-subunit enzyme V-ATPase and is crucial for proton transport [19, 30, 43–45]. Furthermore, we characterized the gene expression patterns of H^+^ transporters to determine whether they are differentially expressed in coral tissues, and we discussed their potential physiological roles. In addition, we investigated the molecular response of *S. pistillata* to ocean acidification by assessing the effect of external seawater acidification on the levels of H^+^ transporter expression after 1 week and 1 year of exposure.

## Results

### Candidate Na^+^-H^+^ exchanger (SLC9) in *S. pistillata*: gene structure, amino acid sequence and phylogenetic analysis

**Fig. 1:**
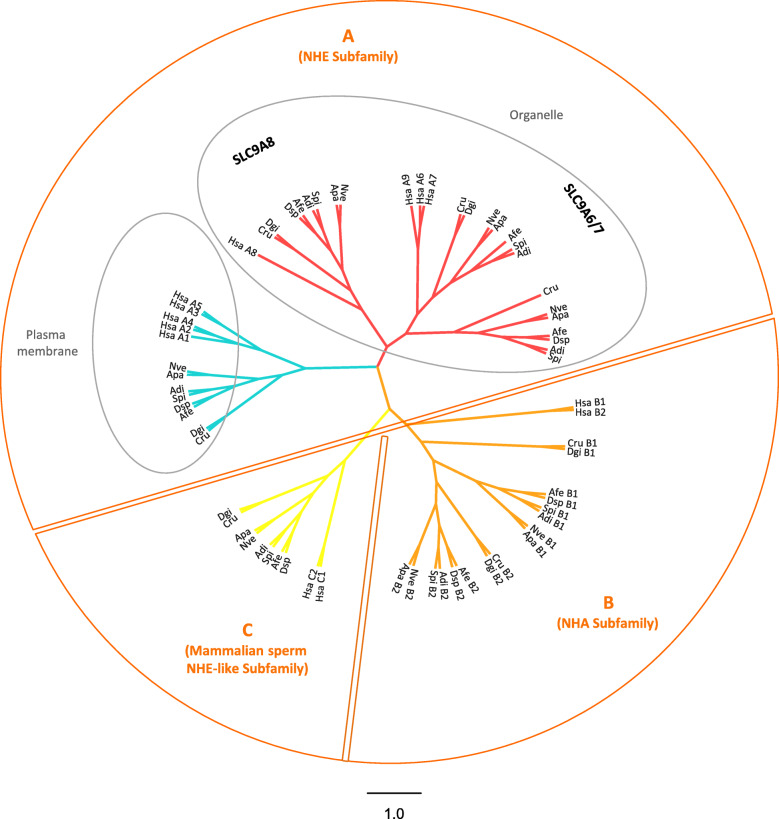
A maximum likelihood phylogenetic (Phyml, LG + I + G) tree of human and cnidarian SLC9s (protein sequences were previously aligned by Clustal Omega). Phylogenetic analysis of *S. pistillata* SLC9 sequences with functionally characterized SLC9s in *H. sapiens* grouped cnidarian SLC9s within three different subfamilies: subfamily A, including plasma membrane and organelle homologs, which are represented in blue and red, respectively; subfamily B, which is represented in orange; and subfamily C, which is represented in yellow. Cnidarian species include *Stylophora pistillata* (Spi), *Acropora digitifera* (Adi), *Nematostella vectensis* (Nve), *Aiptasia pallida* (Apa)*, Corallium rubrum* (Cru)*, Dendronephthya gigantea* (Dgi)*, Amplexidiscus fenestrafer* (Afe)*,* and *Discosoma sp.* (Dsp). Chordata species include *Homo sapiens* (Hsa)

We identified genes homologous to human *SLC9* in the genome and transcriptome of *S. pistillata* [46, 47]. Phylogenetic analysis of *S. pistillata* SLC9 proteins (spiSLC9) with functionally characterized SLC9 in humans allowed us to group the corresponding *S. pistillata* genes within three subfamilies, namely, subfamilies A, B and C (Fig. 1). Members of subfamily A are distributed in two different clusters (plasma membrane and organelle) that contain plasma membrane and organelle homologs, respectively. In addition, organelle homologs include two distinct branches (A8 and A6/7). Of the seven *SLC9* genes identified in *S. pistillata*, four belong to subfamily A (the NHE subfamily), one (*spiSLC9A1*) is a plasma membrane homolog, two (*spiSLC9A6* and *spiSLC9A7*) are A6/7 organelle homologs; one (*spiSLC9A8*) is an A8 organelle homolog; two (*spiSLC9B1* and *spiSLC9B2*) belong to subfamily B (the NHA subfamily); and one (*spiSLC9C*) belongs to subfamily C (the mammalian sperm NHE-like subfamily). The gene and transcript information of SLC9 family members is given in Additional files 1 and 2.

The exchange domain of spiSLC9s is predicted to have 8 to 12 transmembrane segments (TMs) (Additional file 3), which display sequence conservation in contrast to the higher variability observed at the N-termini and C-termini of the proteins. Sequence comparison of spiSLC9s showed similarities between members of the same subfamily, and the percentages of similarity and identity varied for each subfamily. For members of subfamily A, the percentage of similarity varies between 50 and 57% (sharing 35% identity). However, members of subfamily B share 78% similarity (sharing 62% identity). spiSLC9 proteins also exhibit similarity with hSLC9s: spiSLC9A has 42–55% identity and 60–71% similarity to hSLC9A; spiSLC9B has 43–47% identity and 61–66% similarity to hSLC9B; and spiSLC9C has 26% identity and 50% similarity to hSLC9C. In addition, spiSLC9s possess conserved features with hSLC9s, including residues related to Na^+^/H^+^ exchanger activity, such as F161, P167–168, R440 and G455–456, for members of subfamily A (Additional file 4); glycine zipper sequences (SLC9B1-GZ1: 275–283; SLC9B2-GZ1: 184–192; GZ2: 210–216; GZ3: 263–271) for members of subfamily B (Additional file 5); and a conserved voltage-sensing domain (VSD) composed of four transmembrane segments S1-S4 and a cyclic nucleotide-binding domain (CNBD) for members of subfamily C (Additional file 6).

Regarding post-translational modifications, several phosphorylation and N-glycosylation sites were predicted for spiSLC9s (Additional file 7).

### Candidate V_0_ V-ATPase subunit-a in *S. pistillata*: gene structure, amino acid sequence and phylogenetic analysis

**Fig. 2:**
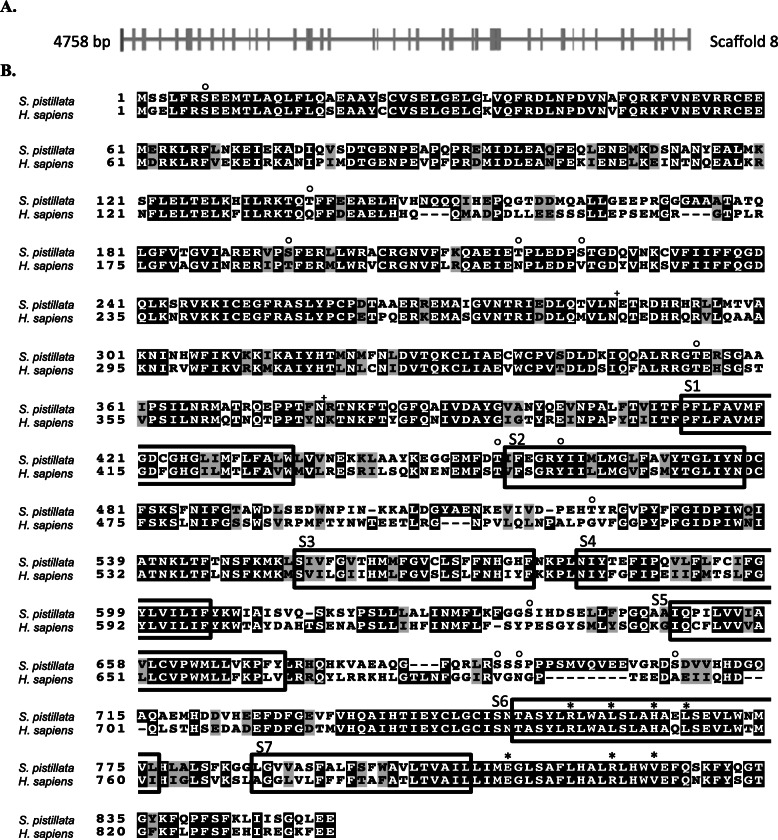
**a** Exon/intron organization of *V*_*0*_ *V-ATPase subunit a* in the genome of *S. pistillata*. Exons are represented as boxes, whereas introns are depicted as lines. **b** Sequence comparison of *S. pistillata* and *H. sapiens* V_0_ V-ATPase subunit a. Identical and similar amino acids (aa) are shaded in black and grey, respectively. The boxes represent the predicted transmembrane segments in *H. sapiens* V_0_ V-ATPase subunit a. The circles and crosses represent phosphorylation and N-glycosylation sites, respectively, in spiH_v_CN1.1 and spiH_v_CN1.2. The asterisk indicates a conserved R relevant to H^+^ transport

As previously described for *spiSLC9*, we identified one gene homologous to human *V*_*0*_ *V-ATPase subunit-a* in the genome and transcriptome of *S. pistillata*. The gene and transcript information of the *V*_*0*_ *V-ATPase subunit-a* gene is given in Fig. 2a and Additional file 2. *spiV*_*0*_ *V-ATPase subunit-a* exists in four splice variants and is one of the 14 subunits composing the spiV-ATPase (Additional file 8).

The spiV_0_ V-ATPase subunit-a protein has six predicted TMs (Additional file 3) and exhibits similarity to human homologs (44–64% similarity and 59–76% identity). Several residues (Fig. 2b) are conserved between spiV_0_ V-ATPase and hV_0_ V-ATPase, such as R735, L739, H743, E789, L746, R799 and V803. Regarding post-translational modifications, several phosphorylation and N-glycosylation sites were predicted for spiV_0_ V-ATPase subunit-a (Additional file 7).

**Fig. 3:**
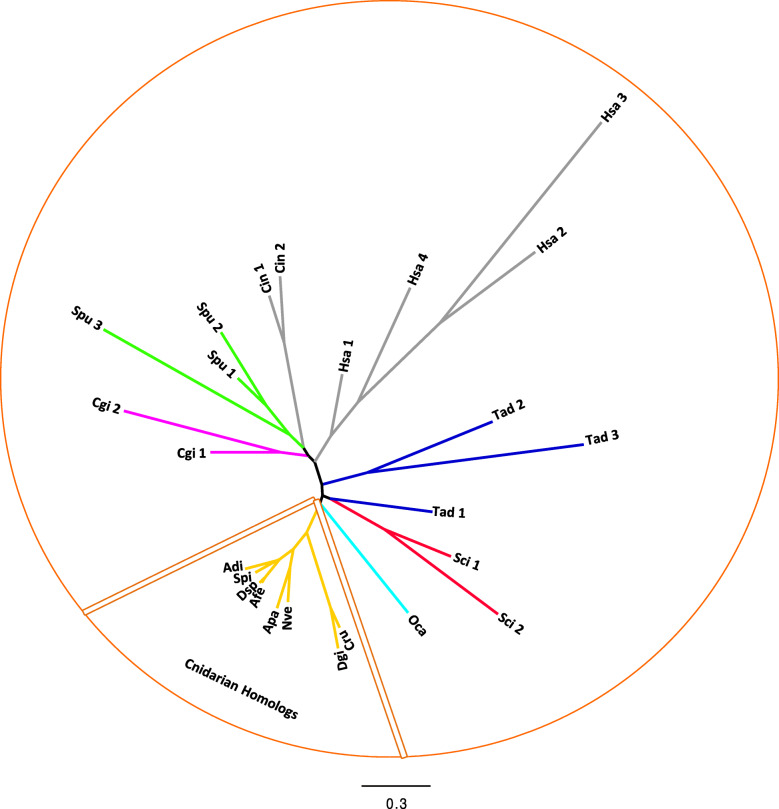
Maximum likelihood (Phyml, LG + I + G) phylogenetic tree of V_0_ V-ATPase subunit-a (protein sequences were previously aligned by Clustal Omega). Cnidarian species include *Stylophora pistillata* (Spi), *Acropora digitifera* (Adi), *Nematostella vectensis* (Nve), *Aiptasia pallida* (Apa)*, Corallium rubrum* (Cru)*, Dendronephthya gigantea* (Dgi)*, Amplexidiscus fenestrafer* (Afe)*,* and *Discosoma sp.* (Dsp). Mollusca species include *Crassostrea gigas* (Cgi). Echinodermata species include *Strongylocentrotus purpuratus* (Spu) and *Acanthaster planci* (Apl). Chordata species include *Homo sapiens* (Hsa) and *Ciona intestinalis* (Cin). Placozoa species include *Trichoplax adhaerens* (Tad). Porifera calcarea species include *Sycon ciliatum* (Sci). Porifera Homoscleromorpha species include *Oscarella carmela* (Oca)

As previously described, we identified putative V_0_ V-ATPase subunit-a homologs in other species (Fig. 3).

### Candidate voltage-gated H^+^ channels (H_v_CN) in *S. pistillata*: gene structures, amino acid sequences and phylogenetic analysis

**Fig. 4:**
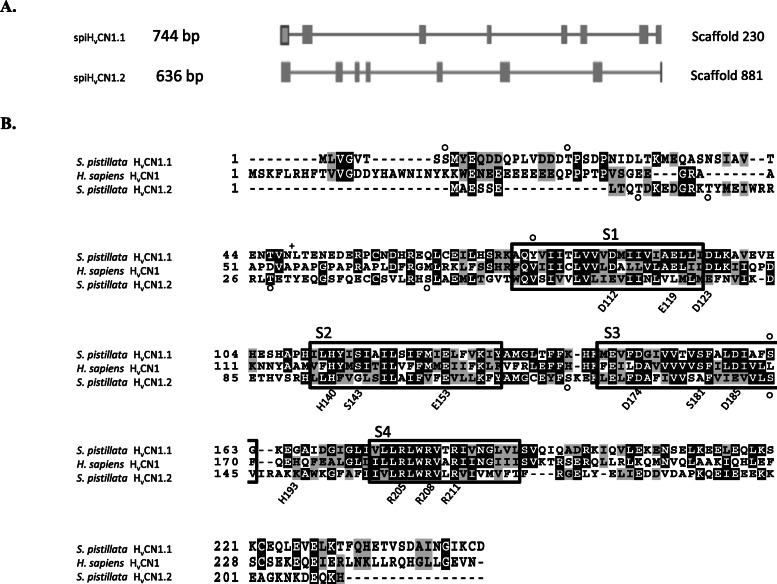
**a** Exon/intron organization of *spiH*_*v*_*CNs* in the genome of *S. pistillata*. Exons are represented as boxes, whereas introns are depicted as lines. **b** Sequence comparison of *S. pistillata* and *H. sapiens* H_v_CN proteins. Identical and similar amino acids (aa) are shaded in black and grey, respectively, whereas aa that are missing from the other sequence are denoted by dashes. The boxes represent the predicted transmembrane segments in human H_v_CN1 (S1-S4). The circles and crosses represent phosphorylation and N-glycosylation sites, respectively, in spiH_v_CN1.1 and spiH_v_CN1.2

Data mining in the genome and transcriptome of *S. pistillata* allowed us to identify two genes, *spiH*_*v*_*CN1.1* and *spiH*_*v*_*CN1.2*, homologous to human *hH*_*v*_*CN1*. The gene and transcript information of the two *spiH*_*v*_*CN* genes is given in Fig. 4a and Additional file 2.

spiH_v_CN1.1 and spiH_v_CN1.2 share 35% identity and 62% similarity and possess four predicted transmembrane domains (TMs) (Additional file 3). The TMs display sequence conservation (Fig. 4b), with highly conserved residues relevant to H_v_CN activity mostly located in the TMs. The two spiH_v_CN sequences exhibit similarity with hH_v_CN1: spiH_v_CN1.1 has 40% identity and 67% similarity to hH_v_CN1, and spiH_v_CN1.2 has 28% identity and 61% similarity to hH_v_CN1. Several basic and acidic residues (R205, R208, R211, H140, E153 and D174) are conserved between spiH_v_CNs and hH_v_CN1, whereas the residues D112, D123, D185 and E119 are conserved only between spiH_v_CN1.1 and hH_v_CN1. Regarding post-translational modifications, several phosphorylation sites were predicted for both spiH_v_CNs, whereas N-glycosylation sites were predicted only for spiH_v_CN1.1 (Additional file 7).

**Fig. 5:**
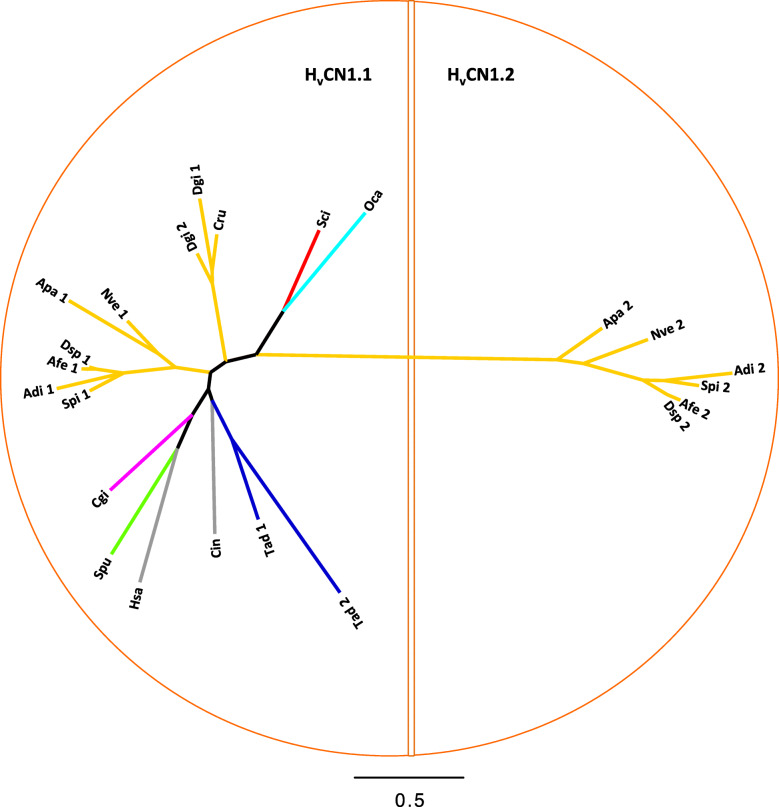
Maximum likelihood (Phyml, LG + I + G) phylogenetic tree of voltage-gated proton channels (protein sequences were previously aligned by Clustal Omega). H_v_CN1.1 and H_v_CN1.2 are separated into two semicircles. Cnidarian species include *Stylophora pistillata* (Spi), *Acropora digitifera* (Adi), *Nematostella vectensis* (Nve), *Aiptasia pallida* (Apa)*, Corallium rubrum* (Cru)*, Dendronephthya gigantea* (Dgi)*, Amplexidiscus fenestrafer* (Afe)*,* and *Discosoma sp.* (Dsp). Mollusca species include *Crassostrea gigas* (Cgi). Echinodermata species include *Strongylocentrotus purpuratus* (Spu) and *Acanthaster planci* (Apl). Chordata species include *Homo sapiens* (Hsa) and *Ciona intestinalis* (Cin). Placozoa species include *Trichoplax adhaerens* (Tad). Porifera calcarea species include *Sycon ciliatum* (Sci). Porifera Homoscleromorpha species include *Oscarella carmela* (Oca)

Sequence similarity searches of available proteomic and genomic datasets using the human H_v_CN1 protein identified putative H_v_CN homologs in several evolutionarily distant species (Fig. 5). Members of Hexacorallia are the only species that possess two H_v_CNs, which splits the tree into two groups: H_v_CN1.1 and H_v_CN1.2.

### Tissue-specific gene expression of *S. pistillata* H^+^ transporters

**Fig. 6:**
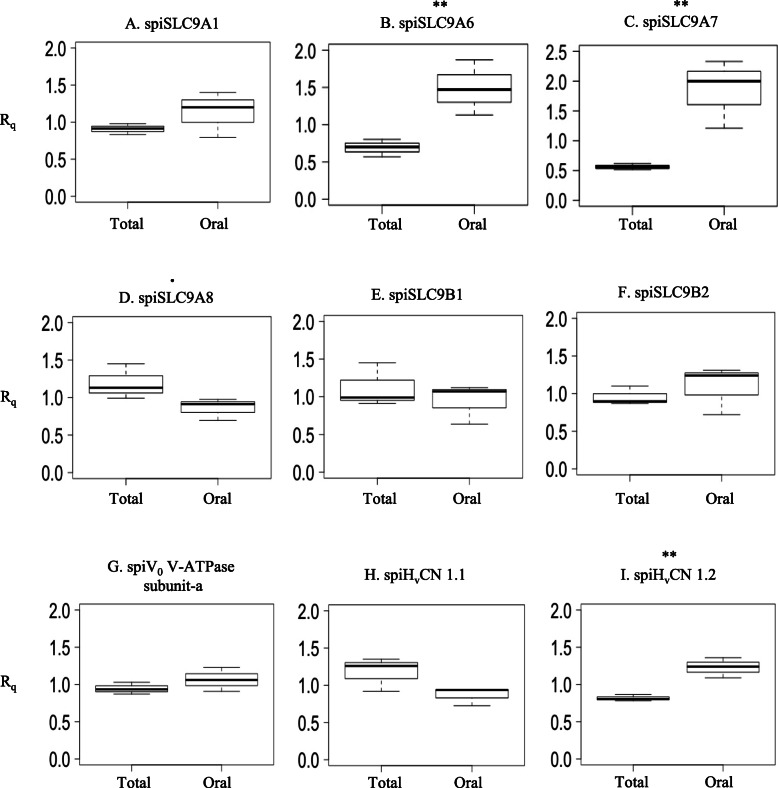
Relative mRNA quantification (R_q_) of *SLC9s, V*_*0*_ *V-ATPase subunit-a* and *H*_*v*_*CNs* measured in total (total colony) and oral (oral fraction) fractions. Box and whisker plots show the first, second (median) and third quartiles (horizontal lines of the boxes) and the respective whiskers (vertical lines spanning the lowest and highest data points of all data, including outliers). The replicate numbers (*n* = 3) represent separate coral samples. The asterisks and points indicate significant differences (• 0.11 ≤ *p*-value≤0.10 and ** *p*-value< 0.05)

Quantitative real-time PCR was used to detect H^+^ transporter mRNA expression (relative mRNA quantification, R_q_) in the oral fraction and the total colony (prepared according to Ganot et al., 2015 and Zoccola et al., 2015) of *S. pistillata*. The results showed no differential expression of *spiSLC9A1*, *spiSLC9B1, spiSLC9B2* or *spiV*_*0*_ *V-ATPase subunit-a* (Fig. 6a, e, f and g and Additional file 9) between the two coral fractions. *spiSLC9A6* and *spiSLC9A7* (Fig. 6b and c) were more highly expressed (*p*-value = 0.023 and 0.018, respectively) in the oral fraction than in the total colony, and *spiSLC9A8* (Fig. 6d) was more highly expressed (*p*-value = 0.108) in the total colony than in the oral fraction. Finally, *spiH*_*v*_*CN1.1* (Fig. 6h) was more highly expressed (*p*-value = 0.107) in the total colony than in the oral fraction, whereas *spiH*_*v*_*CN1.2* (Fig. 6i) was more highly expressed (*p*-value = 0.007) in the oral fraction than in the total colony.

### Effect of ocean acidification on the gene expression of *S. pistillata* H^+^ transporters

**Fig. 7:**
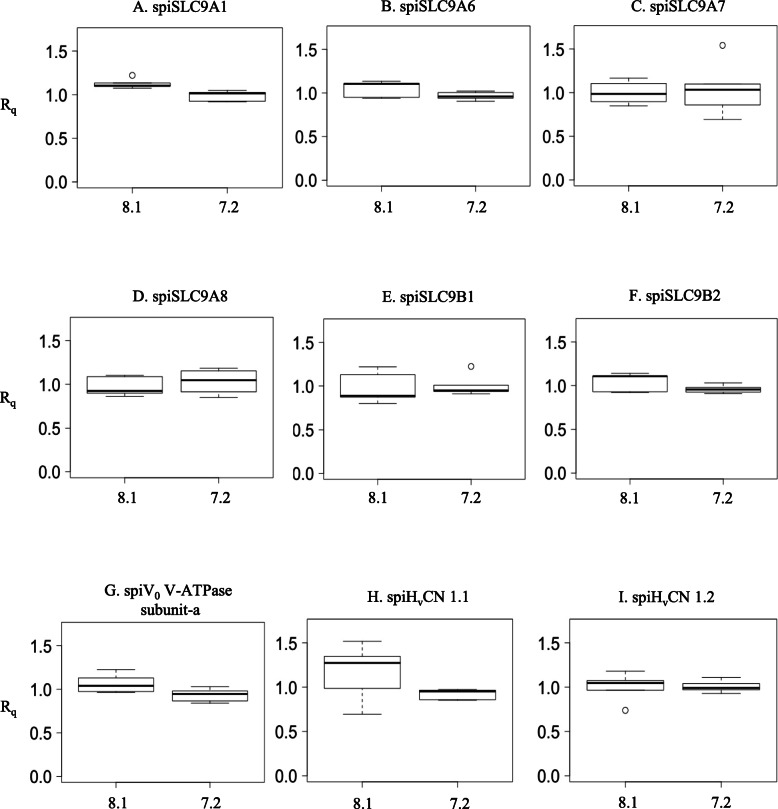
Relative mRNA quantification (R_q_) of *SLC9s, V*_*0*_ *V-ATPase subunit-a* and *H*_*v*_*CNs* at 1 week of *p*CO_2_ exposure plotted against pH 8.1 and 7.2 (*n* = 5)

**Fig. 8:**
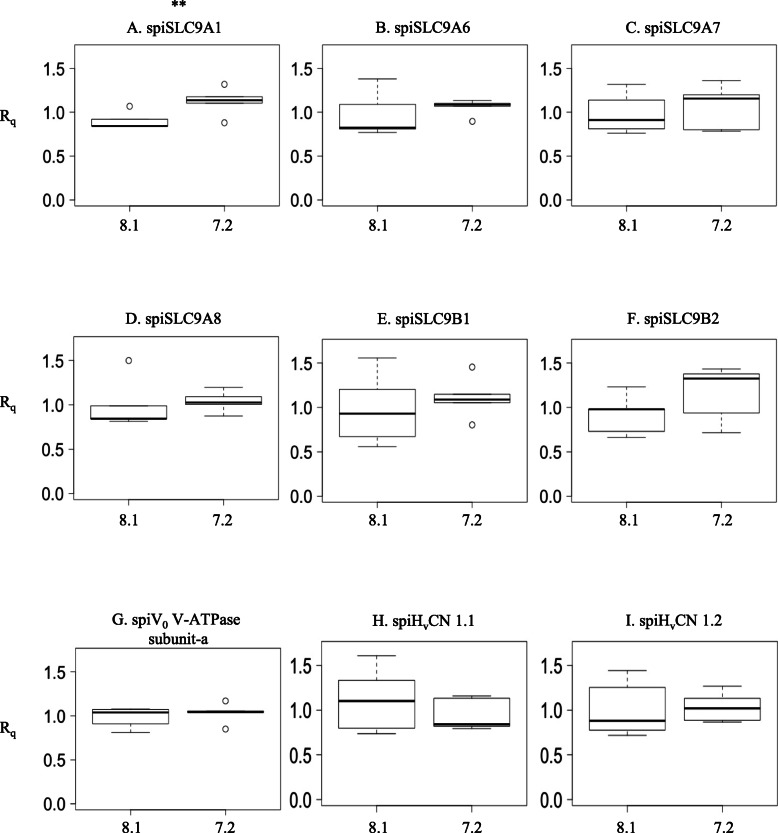
Relative mRNA quantification (R_q_) of *SLC9s, V*_*0*_ *V-ATPase subunit-a* and *H*_*v*_*CNs* at 1 year of *p*CO_2_ exposure plotted against pH 8.1 and 7.2 (*n* = 5). The asterisks indicate significant difference (** *p* < 0.05)

To study the effect of seawater acidification on H^+^ transporter mRNA expression, real-time qPCR analysis of the *spiSLC9A-B, spiV*_*0*_ *V-ATPase subunit-a* and *spiH*_*v*_*CN* genes was carried out in *S. pistillata* micro-colonies exposed to control and acidified seawater (pH 8.1 and 7.2, respectively) for two different durations: 1 week and 1 year. The results showed that there was no difference in the expression of these genes between micro-colonies exposed to pH 8.1 and pH 7.2 for 1 week (Fig. 7 and Additional file 10). After 1 year of exposure to lower pH, there was no difference in the expression of most H^+^ transporter-coding genes (Fig. 8 and Additional file 10). Only *spiSLC9A1* was more highly expressed (*p*-value = 0.029) at pH 7.2 than at the control pH of 8.1 (Additional file 10).

## Discussion

### Phylogeny, domain topology and motif analysis of SLC9s from *S. pistillata*

We identified SLC9 homologs in all anthozoan species (Fig. 1). The anthozoan SLC9 proteins cluster within at least one subfamily (A, B and C), as previously described, suggesting a common ancestor at the time of Bilateria-Radiata separation.

The spiSLC9s characterized in *S. pistillata* show similar characteristics to those of human SLC9s [25, 48], such as a highly homologous transmembrane domain and a variable cytosolic domain. In addition, several key residues important for the function of hSLC9s are also conserved in spiSLC9s. For the A subfamily (Additional file 4), these residues include F161, which is important for Na^+^ transport and acts as a pore-lining residue [49]; P167 and P168, which play a structural role in the folding of the transmembrane domain [50] and influence the targeting and expression of SLC9A proteins [49]; and R440 and G455/G456, which are located in the so-called “glycine rich region” and contribute to the proper functioning of the putative “pH_i_ sensor” domain [51]. For the B subfamily (Additional file 5), the conserved features between the human and *S. pistillata* homologs are especially apparent between glycine zipper (GZ) sequences. GZ sequences mediate close helix-helix folding within transmembrane structures and facilitate the formation of membrane pores. This feature might facilitate access to the mitochondrial inner membrane, where these proteins are highly abundant [52]. Finally, the members of subfamily C (Additional file 6) contain two domains that are conserved with the human homolog: the VSD and the CNBD. The VSD of spiSLC9C carries positively charged residues (K and R) in S4 that are also strongly conserved in the SLC9C of *Strongylocentrotus purpuratus*, *Ciona intestinalis*, *Lepisosteus oculatus* and *Drosophila melanogaster* [53]. Some of these residues are missing in *Homo sapiens*, suggesting that voltage activation differs between these species and humans. The CNBD also regulates the activity of spiSLC9C, probably through the binding of cyclic adenosine monophosphate (cAMP) produced by the soluble adenylyl cyclase enzyme (sAC), as reported in the sea urchin [53].

Overall, the conserved features of spiSLC9s align with their roles as Na^+^/H^+^ exchangers. Their shared properties with previously characterized SLC9s indicate that they retain similar functions and provide insights into their activation mechanism and regulation in *S. pistillata*.

### Phylogeny, domain topology and motif analysis of V_0_ V-ATPase subunit-a from *S. pistillata*

We identified V_0_ V-ATPase subunit-a homologs in Anthozoa (Fig. 3). In contrast to most species, which possess more than one V_0_ V-ATPase subunit-a homolog, anthozoans possess only one. This suggests that the specialization of V_0_ V-ATPase subunit-a homologs could be phylum-specific or even species-specific [54].

V_0_ V-ATPase subunit-a in *S. pistillata* shares similar characteristics with those in yeast and humans, including the number of predicted TMs, which falls within the TM range of its homologs (5–8 TMs) [55]. TMs are thought to form proton-conducting hemichannels that allow H^+^ to translocate across the membrane [56]. Previous studies in yeast allowed the identification of functional residues in V_0_ V-ATPase subunit-a through the use of random and site-direct mutagenesis. Among these residues, R735 is known to play an essential role in proton transport, as mutations of this residue result in complete inactivation of the ATP-dependent H^+^ transport of the V-ATPase [26]. The conservation of this residue in *S. pistillata* suggests that R735 fulfils its role in H^+^ transport (Fig. 2b). Other residues (L739, H743, L746, E789, R799 and V803) involved in proton translocation and ATPase activity [55] are also conserved in *S. pistillata*.

Overall, the features conserved between spiV_0_ V-ATPase subunit-a and its human and yeast homologs align with its role as the subunit-a of the V_0_ V-ATPase. Furthermore, the identification of homologs of all the V-ATPase subunit-encoding genes in the genome and transcriptome of *S. pistillata* (Additional file 8) suggests a conserved organization between the *S. pistillata* V-ATPase and the human V-ATPase.

### Phylogeny, domain topology and motif analysis of H_v_CNs from *S. pistillata*

One H_v_CN family member (H_v_CN1.1) was identified in all species examined in this study, including the four anthozoan orders, namely, Actinaria, Alcyonacea, Corallimorpharia and Scleractinia (Fig. 5). In addition, we report for the first time a second member of the H_v_CN family (H_v_CN1.2) in some Cnidaria. Genomic and transcriptomic searches of H_v_CN1.2 in public databases of Octocorallia and Hydrozoa (*Hydra magnipapillata*) did not produce any results (data not shown). The selective expression of H_v_CN1.2 only in Hexacorallia suggests that it is specific to this cnidarian subclass, and its presence in noncalcifying anthozoans (Corallimorphs and Actinaria) suggests that it is not linked to the appearance of aragonite biomineralization in Scleractinia [57]. The two voltage-gated proton channels (spiH_v_CNs) characterized in *S. pistillata* are highly divergent. This finding is further supported by the length of the phylogenetic branch (Fig. 5) that separates the two homologs: the amino acid similarity between spiH_v_CN1.1 and hH_v_CN1.1 is higher than that between spiH_v_CN1.1 and spiH_v_CN1.2.

The spiH_v_CNs possess molecular properties that are hallmarks of all H_v_CNs, such as the four transmembrane segments, the basic (R) and acidic residues (D and E) associated with voltage sensing, and the coiled-coil structure at the C-terminus [58]. The gating of proton channels is tightly regulated by pH and voltage, ensuring that they open only to extrude H^+^ from the cell [43]. Phosphorylation of the spiH_v_CNs might activate these channels and enable them to open faster and at less positive voltages than those required without activation, as previously reported in human leukocytes [59–62].

SpiH_v_CN1.1 and spiH_v_CN1.2 share similar structures and organizations (Fig. 4). However, differences identified at the protein sequence level might reflect some unique features. First, many acidic residues, which are known to be associated with voltage sensing in hH_v_CN1 [58, 63], are present in spiH_v_CN1.1 but not in spiH_v_CN1.2. This could result in a different sensitivity to voltage, with spiH_v_CN1.2 being less sensitive than spiH_v_CN1.1. Additionally, among these residues, D112 in S3, which is known to be essential for proton and charge selectivity [61], is missing and replaced by E112 in spiH_v_CN1.2. However, a previous study demonstrated that the substitution of D112 with an acidic residue in the same position maintained proton specificity in hH_v_CN1 [61], suggesting that both spiH_v_CNs are proton specific. Another difference concerns the Zn^+ 2^-binding residues of spiH_v_CNs. In humans and mice, proton currents are suppressed by extracellular Zn^+ 2^, which binds to four Zn^+ 2^-coordinating residues (E119, D123, H140 and H193) [64, 65]. By sequence comparison, we observed that at these positions, some residues are not conserved in spiH_v_CN1.1 (H193G) and are even less conserved in spiH_v_CN1.2 (E119L, D123E and H193K). Hence, we suggest that the replacement of Zn^+ 2^-coordinating residues with other residues potentially affects the Zn^+ 2^ sensitivity of spiH_v_CNs, as reported in vertebrates [66, 67]. Since spiH_v_CN1.1 contains more Zn^+ 2^-coordinating residues, we propose that it is more sensitive to Zn^+ 2^ than spiH_v_CN1.2.

Overall, the conserved features of the spiH_v_CNs align with their roles as voltage gated H^+^-channels. Common properties between the spiH_v_CNs and hH_v_CN preserve their voltage sensitivity and proton specificity, with some differences concerning their Zn^+ 2^ sensitivity. In addition, distinctive properties between different spiH_v_CNs suggest differential regulation, possibly linked to their localization/function. Future analyses, however, are required to validate these assumptions and provide insight into their physiological role.

### Tissue-specific expression patterns of H^+^ transporter genes in *S. pistillata*

Whole coral colonies are typically used in conventional techniques of gene expression analysis, limiting the possibility of further differentiating specific gene expression between oral and aboral tissues. These tissues contain different cell subtypes; some of them are more abundant (e.g., endosymbiotic dinoflagellates) or exclusively found (e.g., cells specialized for food digestion and reproduction, nematocysts) in the oral tissue, whereas others are exclusively found in the aboral tissue (e.g., calcifying cells) (Veron et al., 1993; Peter et al., 1997) [68]. As several physiological functions are associated with these cellular subtypes, analysing the differential gene expression of H^+^ transporters in the two coral tissues helps identify the potential physiological processes in which they might participate.

To perform this task, we used a previously developed micro-dissection protocol [20, 69] to separate the oral fraction (including the oral disc and most of the polyp body, with no or minimal contamination of cells from the aboral tissue) from the total colony. We then compared the expression of H^+^ transporters in the oral fraction to that in the total colony.

Our results demonstrate that some H^+^ transporters are more highly expressed in the oral fraction than in the total colony (hereafter referred to as “oral-specific”); others are more highly expressed in the total colony than in the oral fraction (hereafter referred to as “aboral-specific”); and others are expressed at the same levels in both fractions (hereafter referred to as “ubiquitous”) (Fig. 6). Using H^+^ transporter tissue expression and the existing literature in other systems as supporting information, we discuss the role that these transporters might play in corals.

The oral-specific H^+^ transporters include spiSLC9A6, spiSLC9A7 and spiH_v_CN1.2. spiSLC9A6 and spiSLC9A7 are organellar homologs (Fig. 1), and as reported for humans [48, 70], they might play a role in vesicular neurotransmitter uptake in oral polyps, where an elaborate nerve ring system is present [71]. In addition, cells in the oral tissue are enriched with zooxanthellae [72], which produce and incorporate high levels of anionic superoxide (e.g., throughout photosynthesis) and Zn^+ 2^ (e.g., through uptake from seawater) that accumulate in the host cytoplasm [73–75]. Thus, spiH_v_CN1.2 might favour the exit of H^+^ at the basolateral membrane of these cells, as reported in human osteoblasts [76], preventing the membrane depolarization to extreme negative voltages associated with O_2_^−^ electron transfer. Additionally, the decreased Zn^+ 2^ sensitivity of spiH_v_CN1.2 compared to that of spiH_v_CN1.1 (see Discussion 3.1.3) could be correlated with the higher Zn^+ 2^ concentration levels in the oral (symbiotic) cells [67, 77–80].

The aboral-specific H^+^ transporters are spiH_v_CN1.1 and spiSLC9A8. These transporters could play a role in the pH_i_ regulation of calcifying cells. During the calcification reaction, H^+^ is produced in the ECM and needs to be removed to promote an alkaline environment favourable for calcification [11, 81]. The Ca^+ 2^-ATPase at the apical side of the calcifying cells has been suggested to be involved in pumping out H^+^ from the ECM [41]. On the basal side of the cell membrane, spiH_v_CN1.1 might contribute to extruding excess H^+^ from the cytoplasm of calcifying cells, similar to the role it plays in coccolithophores [82]. Finally, the organellar spiSLC9A8 might regulate medial/trans-Golgi pH and intracellular trafficking, as in humans [83, 84], since in calcifying cells, this function is particularly necessary for the regulation of organic matrix synthesis and secretion [85].

**Fig. 9:**
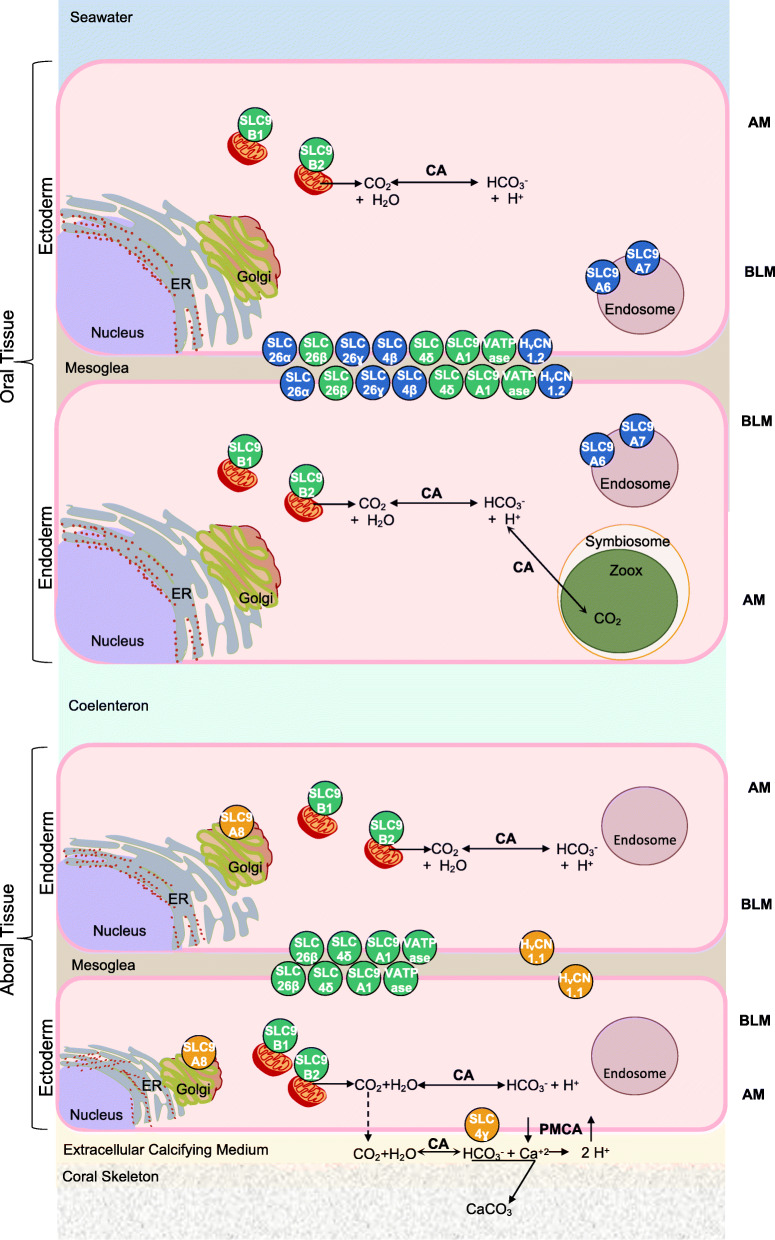
Model of acid-base transporters involved in intracellular pH regulation expressed on the apical (AM) and basolateral (BLM) membranes of coral cells throughout the tissue layers. The roles of other ion channels and transporters involved in other cellular processes are not considered here. Transporters that are more highly expressed in the oral fraction (oral-specific) are coloured in blue, those that are more highly expressed in the total colony (aboral-specific) are coloured in orange, and those that are expressed at the same levels in both fractions (ubiquitous) are coloured in green. Other enzymes (CA = carbonic anhydrase) and transporters (PMCA = Ca^+ 2^ ATPase) involved in the H^+^ flux balance are represented in bold letters

The rest of the H^+^ transporters (V_0_ V-ATPase subunit-a, spiSLC9A1, spiSLC9B1 and spiSLC9B2) are ubiquitous. Our results suggest that the coral V_0_ V-ATPase subunit-a homolog is ubiquitously expressed and differs from those identified in humans. Indeed, the human V_0_ V-ATPase subunit-a isoforms have different tissue distributions with intracellular or apical/basolateral membrane localization [31, 55, 86–89]. This difference is probably linked to the primers used for the detection of the coral V_0_ V-ATPase subunit-a, which do not discern the different isoforms (X1, X2, X3 and X4; see Additional file 8) but recognize all of them. We suggest that other ubiquitous transporters play housekeeping roles. spiSLC9A1, for example, could play a role in homeostatic pH regulation on the basolateral cell membrane, and spiSLC9B1–2 might participate in organismal ion homeostasis on the mitochondrial inner membrane, as reported in vertebrates [19, 90–93]. Interestingly, spiSLC9A1 was the only transporter affected by seawater acidification after 1 year of exposure (Fig. 8). Its activation might be triggered by the pH_i_ sensor domain (see Discussion 3.1.1), which activates the transporter at low pH_i_ values, similar to humans [19]. Figure 9 summarizes the tissue distribution of the principal acid-base transporters that could participate in the intracellular pH regulation of coral cells based on the results obtained by performing real-time PCR of oral fraction and total colony coral samples. These transporters include the H^+^ transporters SLC9, V-ATPase and H_v_CN, which were characterized in the present study, and the HCO_3_^−^ transporters SLC4 and SLC26, which were characterized by Zoccola et al. in 2015.

## Conclusions

This study provides the first molecular characterization in the coral *S. pistillata* of several families of H^+^ transporters*,* which are known as pH_i_ regulators in animal cells. Most importantly, we report for the first time a novel member of the H_v_CN gene family, H_v_CN1.2, in the cnidarian subclass Hexacorallia. The identification of conserved residues between coral and human H^+^ transporter homologs suggests functional conservation related to intracellular pH_i_ regulation.

However, additional experiments (e.g., gene silencing experiments, pharmacological experiments using inhibitors, electrophysiological measurements of H^+^ currents, etc.) with a larger number of replicates need to be carried out in the future to demonstrate the participation of these H^+^ transporters in coral acid-base cellular homeostasis. Moreover, we assessed the tissue specificity of the H^+^ transporter gene families in the coral *S. pistillata,* and we observed that their expression was not restricted to only one specific tissue (oral or aboral), as reported for some members of the HCO_3_^−^ gene family [20]. However, we observed higher or lower expression profiles in the oral or aboral tissues. These results both highlight the importance of H^+^ transporters in the coral colony and suggest that they take part in homeostatic (e.g., intracellular acid-base balance) and physiological processes (e.g., calcification, photosynthesis, food digestion). Finally, we investigated the impact of OA on H^+^ transporter gene expression in *S. pistillata,* and we identified one candidate gene (*spiSLC9A1*) involved in the coral response to ocean acidification that showed differential expression after long-term exposure to acidified seawater (1 year). The other H^+^ transporters did not show any significant changes at the gene level under seawater acidification conditions. Nevertheless, the modulation of gene expression can also occur at the protein level, which should be investigated in future studies. The influence of other environmental factors, e.g., temperature, remains to be tested and will enrich the understanding of coral phenotypic plasticity. This knowledge is especially relevant to our understanding of the ability of benthic animals to buffer the impacts of environmental changes, thereby providing more time for genetic adaptation to occur. In addition, responses to environmental changes are often species-specific [9], with physiological differences that can potentially reflect a different profile of H^+^ transporter gene expression, which could also be investigated in other coral species. Such comparative studies might aid in the development of molecular markers linked to pH_i_ tolerance traits in coral reef populations.

## Methods

### Biological materials

Experiments were conducted on the symbiotic scleractinian coral *Stylophora pistillata* grown in the long-term culture facilities at the Centre Scientifique de Monaco in aquaria supplied with seawater from the Mediterranean Sea (exchange rate 2% h − 1) under the following controlled conditions: semi-open circuit, temperature of 25 °C, salinity of 38, light exposure of 200 μmol photons m^− 2^ s^− 1^, and a 12 h:12 h light:dark cycle. Samples were prepared from one mother colony as nubbins suspended on monofilament threads. After 3 weeks of cicatrisation, the first set of samples (*n* = 3) was used for the oral fraction micro-dissection experiment, and another set (*n* = 20) was used for the exposure to seawater acidification experiment.

### Exposure to seawater acidification

The seawater acidification setup was performed as described previously [9, 94, 95]. Briefly, carbonate chemistry was manipulated by bubbling with CO_2_ to reduce the pH from the control value of pH 8.1 to the target value of 7.2. Twenty coral nubbins were randomly distributed among four experimental tanks (*n* = 5 for each experimental tank): two tanks (pH 8.1 and 7.2) were reserved for 1 week of exposure, and two were reserved for 1 year of exposure. The experiments were repeated three times to ensure reliability. Similar results were obtained for each experiment (not shown).

### Data mining

The amino acid sequences of human H^+^ transporters were retrieved from NCBI (http://www.ncbi.nlm.nih.gov/protein) and used as a bait to mine (BLAST) the transcriptome, genome and EST databases of the following phyla: Cnidaria (*Stylophora pistillata*, *Acropora digitifera*, *Nematostella vectensis*, *Aiptasia pallida*, *Amplexidiscus fenestrafer*, *Discosoma sp.*, *Corallium rubrum* and *Dendronephthya gigantea*), Mollusca (*Crassostrea gigas*), Echinodermata (*Strongylocentrotus purpuratus*), Chordata (*Ciona intestinalis*), Porifera (*Sycon ciliatum*) and Placozoa (*Trichoplax adhaerens*). The following web servers were used: NCBI (http://www.ncbi.nlm.nih.gov/), EnsemblMetazoa (https://metazoa.ensembl.org/), ReefGenomics (http://reefgenomics.org) and Cnidarian Database (http://data.centrescientifique.mc/).

### Sequence analysis

Putative transmembrane helices in proteins were predicted by the Center for Biological Sequence Analysis Prediction Server (http://www.cbs.dtu.dk/services/) using the TMHMM algorithm. On the same platform, phosphorylation and N-glycosylation prediction analyses were performed using NetPhos and NetNGlyc.

### Phylogenetic analyses

Alignment of H^+^ transporter amino acid sequences was performed using Clustal Omega on the EMBL-EBI server (https://www.ebi.ac.uk/). Based upon the produced amino acid alignments, maximum likelihood estimates of the topology and the branch length were obtained using PhyML v3.1 [96]. The model of substitution used in this step was LG + G, which was previously selected over others via alignment analysis with ProtTest3.4.2 [97]. Phylogenetic trees were then edited using FigTree v1.4.4 (Rambaut et al., 2014).

### Oral fraction micro-dissection

Coral micro-dissection was performed as indicated previously [20, 69]. Briefly, coral nubbins (*n* = 3) were set to rest in a glass petri dish filled with seawater and tricaine mesylate. Once polyps were extended, oral fractions (including the oral disc and most of the polyp body) were cut from the coral colony (total colony) using micro-dissection scissors under a binocular microscope. Both fractions (oral fraction and total colony) were then used for RNA extraction.

### Real-time PCR (qPCR)

Total RNA extraction and cDNA synthesis were performed as described previously [98]. Briefly, RNA was isolated from biological triplicates that underwent micro-dissection or from quintuplicates collected for each pH treatment using an RNeasy kit (Qiagen) according to the manufacturer’s instructions. Reverse transcription was then performed using SuperScript IV Reverse Transcriptase (Invitrogen) on 2 μg of RNA. Thermo cycler conditions were set as follows: 50 min at 50 °C, 7 min at 25 °C, 50 min at 50 °C and 5 min at 85 °C. qPCR runs were performed in 96-well plates on a QuantStudio 3 (Applied Biosystems) machine using PowerUpTM SYBRTM Green Master Mix for PCR amplification. The primer sequences used are provided in Additional file 11. Relative expression was calculated using Biogazelle qBase + 2.6TM [99]. Gene expression was normalized (relative mRNA quantification, Rq) to the expression of two reference genes, ubiquitin-60S ribosomal protein (L40) [100] and an acidic ribosomal phosphoprotein P0 (36B4) [101], after these genes were determined to have acceptably low M values and coefficients of variation [99].

### Statistical analysis

Statistical analysis was performed using R v3.5.2 software. For the oral fraction micro-dissection experiment, a fixed number of samples (*n* = 3) was used due to technical limitations. For the exposure to seawater acidification experiment, a sample size estimation was performed, and *n* = 5 samples were used. For both experiments, the normal distribution of the data was evaluated using Shapiro-Wilk’s test. Samples with a standard deviation ≥5 were excluded from the analysis. This was the case for *spiSLC9C*, whose qPCR results showed great variability, probably due to its extremely low expression values. A t-test was used to identify differentially expressed genes between *S. pistillata* fractions (oral fraction and total colony) and pH treatments (pH 8.1 and pH 7.2). We considered 0.10 ≤ *p*-values ≤0.11to indicate near-marginal significance (•); *p*-values ≤0.1 as significant (**); and *p*-values ≤0.05 as highly significant (*).

## Supplementary Information

**Additional file 1. **Exon/intron organization of *SLC9s* in the genome of *S. pistillata*.

**Additional file 2. **Gene and transcript information of H^+^ transporter family members in *S. pistillata*.

**Additional file 3. **Transmembrane segment prediction (TMs) of H^+^ transporter proteins in *S. pistillata*.

**Additional file 4. **Sequence comparison of the *H. sapiens* SLC9A1 and *S. pistillata* SLC9A proteins.

**Additional file 5. **Sequence comparison of the *S. pistillata* and *H. sapiens* SLC9B proteins.

**Additional file 6. **Sequence comparison of the *S. pistillata* and *H. sapiens* SLC9C1 proteins. The boxes represent human voltage-sensing domains (S1-S4), and the asterisks indicate conserved positively and negatively charged residues relevant to voltage sensing. The rectangles indicate conserved positively charged residues in S4 that are present in *S. pistillata* but missing in hSLC9C1. The triangles indicate residues involved in the cyclic nucleotide-binding domain.

**Additional file 7. **Number of phosphorylation sites and N-glycosylation sites predicted for *S. pistillata* H^+^ transporters.

**Additional file 8. **A. V_0_ V-ATPase subunit-a isoforms in coral *S. pistillata.* B. Human and coral homologs of V-ATPase subunits.

**Additional file 9. **Statistical analysis of the relative mRNA quantification (R_q_) of *SLC9s, V*_*0*_ *V-ATPase subunit-a* and *H*_*v*_*CNs* (*N* = 3) in *S. pistillata* total (total colony) and oral (oral fraction) fractions.

**Additional file 10. **Statistical analysis of the relative mRNA quantification (R_q_) of *SLC9s, V*_*0*_ *V-ATPase subunit-a* and *H*_*v*_*CNs* (*n* = 5) at pH 8.1 and 7.2 after 1 week and 1 year of *p*CO_2_ exposure. The asterisks indicate significant differences (** *p* < 0.05).

**Additional file 11. **List of *S. pistillata* H^+^ transporter genes with RT-PCR primers and product sizes.

## Data Availability

All data needed to evaluate the conclusions in the paper are present in the manuscript and/or the Additional Files. Additional data related to this manuscript are available from the corresponding author on reasonable request. Genomic and transcriptomic data were obtained from the publicly available database of the National Center for Biotechnology Information (https://www.ncbi.nlm.nih.gov/) or from the private database of the Centre Scientifique de Monaco (http://data.centrescientifique.mc/).

## Abbreviations

CNBD: Cyclic nucleotide-binding domain
CPA1: Cation proton antiporter 1
CPA2: Cation proton antiporter 2
ECM: Extracellular calcifying medium
GZ: Glycine zipper
H_v_CN: Voltage-gated proton channel
NaT-DC: Na-transporting carboxylic acid decarboxylase
OA: Ocean acidification
sAC: Soluble adenylyl cyclase
SLC9: Solute carrier 9
TM: Transmembrane segment
V-ATPase: Vacuolar-type electrogenic H^+^-ATP hydrolase
VSD: Voltage-sensing domain
