# Supplementary material for: Intracellular pH regulation: characterization and functional investigation of H+ transporters in Stylophora pistillata
Source: BMC Mol Cell Biol. 2021 Mar 8;22:18. doi: 10.1186/s12860-021-00353-x (PMC7941709; doi:10.1186/s12860-021-00353-x)
Supplement: Supplementary file 1 — Additional file 1. Exon/intron organization of SLC9s in the genome of S. pistillata. [file 12860_2021_353_MOESM1_ESM.pdf]

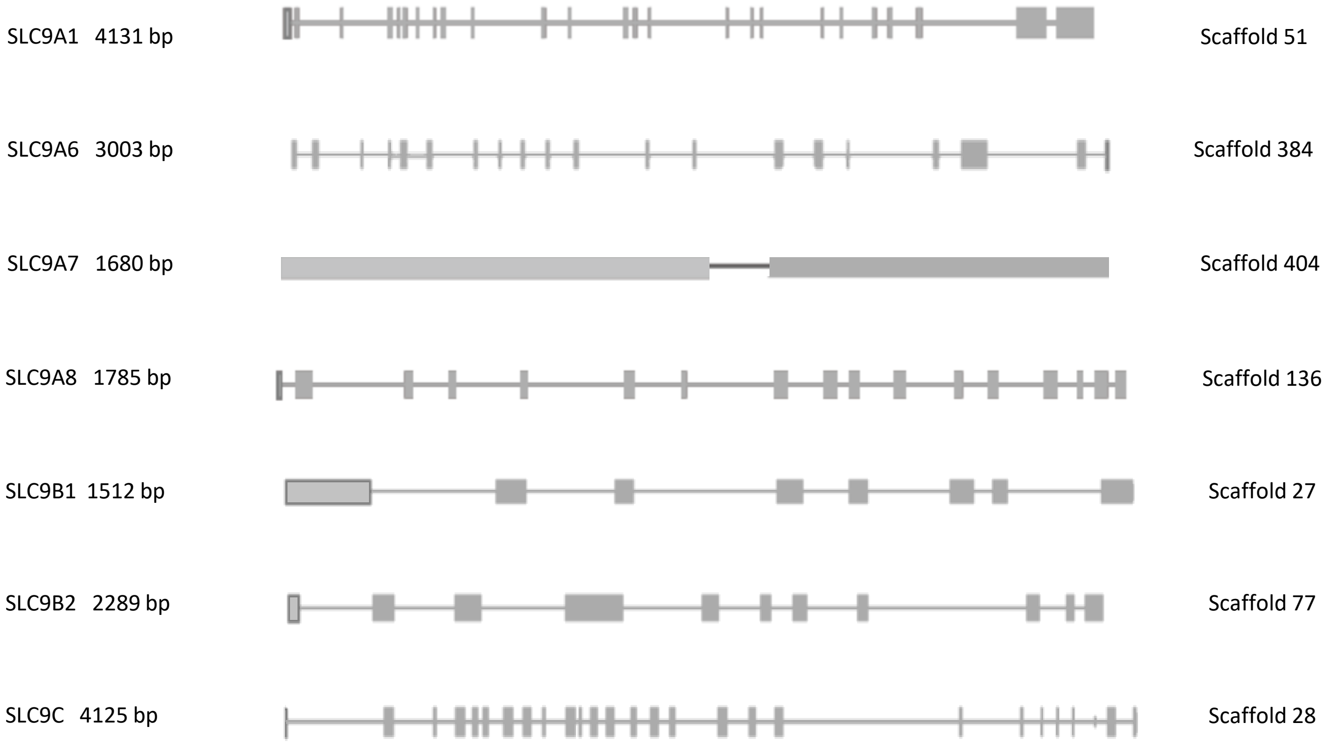
SLC9A1 4131 bp
Scaffold 51
SLC9A6 3003 bp
Scaffold 384
SLC9A7 1680 bp
Scaffold 404
SLC9A8 1785 bp
Scaffold 136
SLC9B1 1512 bp
Scaffold 27
SLC9B2 2289 bp
Scaffold 77
SLC9C 4125 bp
Scaffold 28
