# Supplementary material for: Intracellular pH regulation: characterization and functional investigation of H+ transporters in Stylophora pistillata
Source: BMC Mol Cell Biol. 2021 Mar 8;22:18. doi: 10.1186/s12860-021-00353-x (PMC7941709; doi:10.1186/s12860-021-00353-x)
Supplement: Supplementary file 2 — Additional file 2. Gene and transcript information of H+ transporter family members in S. pistillata. [file 12860_2021_353_MOESM2_ESM.pdf]

Gene	Contig Name[1]	Gene Length – base pairs (bp)	Number of exons	Contig Location	mRNA Length (bp)	mRNA accession number	Protein Length and Weight
SLC9A1	Scaffold_51	1039750	25	399434-374799	4131	[1]	830 aa - 93.5 kDa
SLC9A6	Scaffold_384	313573	20	42651-17078	3003	[1]	647 aa - 72.6 kDa
SLC9A7	Scaffold_404	297488	2	123329-125113	1680	[1]	594 aa - 67.05 kDa
SLC9A8	Scaffold_136	641450	17	505220-496092	1785	XM_022934279.1[2]	594 aa - 65.5 KDa
SLC9B1	Scaffold_27	1282297	8	1273573-1268503	1512	XM_022924097.1[2]	503 aa - 53.9 kDa
SLC9B2	Scaffold_77	865837	11	128552-137576	2289	[1]	515 aa - 55.4 kDa
SLC9C	Scaffold_28	1278021	27	1154858-1179640	4125	[1]	1213 aa - 135.7 kDa
V_0 V-ATPase subunit-a	Scaffold_8	1762381	42	1117835-1152572	4758	XM_022939359.1[2]	853 aa - 97.4 kDa
H_vCN 1.1	Scaffold_230	479899	8	310203-304834	744	XM_022939457.1[2]	247 aa - 27.9 kDa
H_vCN 1.2	Scaffold_881	95377	8	79953-75631	636	XM_022952789.1[2]	211 aa - 24.7 kDa

[1]Data Centre Scientifique de Monaco
[2]NCBI
