# Supplementary material for: Intracellular pH regulation: characterization and functional investigation of H+ transporters in Stylophora pistillata
Source: BMC Mol Cell Biol. 2021 Mar 8;22:18. doi: 10.1186/s12860-021-00353-x (PMC7941709; doi:10.1186/s12860-021-00353-x)
Supplement: Supplementary file 3 — Additional file 3. Transmembrane segment prediction (TMs) of H+ transporter proteins in S. pistillata. [file 12860_2021_353_MOESM3_ESM.pdf]

Protein	TMs	
	Number	Position
**SLC9A1**	12	61-83, 96-115, 125-142, 154-176, 186-208, 217-239, 254-276, 288-307, 311-330, 343-356, 375-397, 443-462
**SLC9A6**	12	5-22, 49-68, 75-97, 150-172, 185-207, 252-274, 295-317, 340-362, 383-405, 415-437, 449-468, 483-505
**SLC9A7**	12	12-34, 54-76, 169-186, 201-223, 235-254, 311-333, 338-360, 365-387, 400-422, 432-454, 461-483, 498-520
**SLC9A8**	11	87-105, 115-137, 146-165, 180-202, 215-237, 289-311, 337-359, 379-398, 405-427, 442-462, 471-493
**SLC9B1**	10	26-48, 63-85, 92-114, 182-204, 232-254, 258-277, 290-324, 339-361, 368-390, 441-463
**SLC9B2**	11	47-69, 74-96, 101-120, 166-188, 193-215, 228-250, 270-289, 302-324, 354-373, 380-402, 453-475
**SLC9C**	8	36-58, 70-92, 138-160, 181-203, 223-245, 257-279, 294-316, 321-343
**V_0 V-ATPase subunit-a**	6	416-438, 459-476, 555-577, 590-612, 648-667, 786-808
**H_vCN 1.1**	4	76-95, 114-138, 145-162, 171-193
**H_vCN 1.2**	4	58-77, 92-110, 127-146, 155-174
