# Supplementary material for: Intracellular pH regulation: characterization and functional investigation of H+ transporters in Stylophora pistillata
Source: BMC Mol Cell Biol. 2021 Mar 8;22:18. doi: 10.1186/s12860-021-00353-x (PMC7941709; doi:10.1186/s12860-021-00353-x)
Supplement: Supplementary file 4 — Additional file 4. Sequence comparison of the H. sapiens SLC9A1 and S. pistillata SLC9A proteins. [file 12860_2021_353_MOESM4_ESM.pdf]

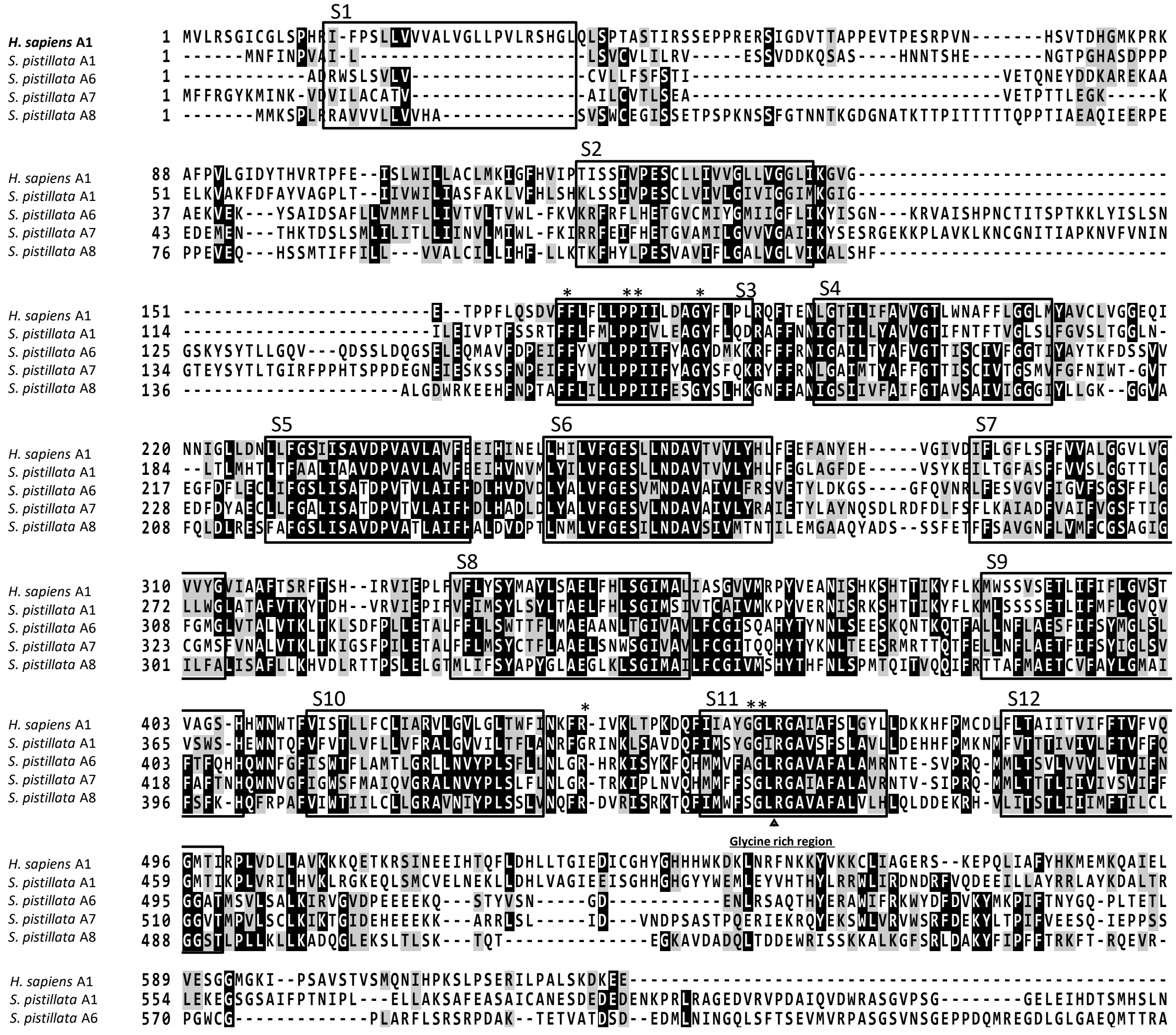
S1
H. sapiens A1 1 MVLRSGICGLSPHRI-FPSLLVVVALVGLLPVLRSHGLQLSPTASTIRSSEPPRERSIGDVTTAPPEVTPESRPVN-------HSVTDHGMKPRK
S. pistillata A1 1 ------MNFINPVAI-L---------------------LSVCVLILRV-----ESSVDDKQSAS----HNNTSHE-------NGTPGHASDPPP
S. pistillata A6 1 ------------ADRWSLSVLV-----------------CVLLFSFSTI------------------------------VETQNEYDDKAREKAA
S. pistillata A7 1 MFFRGYKMINK-VDVILACATV-----------------AILCVTLSEA------------------------------VETPTTLEGK-----K
S. pistillata A8 1 -------MMKSPLRRAVVVLLVVHA-------------SVSWCEGISSETPSPKNSSFGTNNTKGDGNATKTTPITTTTTQPPTIAEAQIEERPE
S2
H. sapiens A1 88 AFPVLGIDYTHVRTPFE--ISLWILLACLMKIGFHVIPTISSIVPESCLLIVVGLLVGGLIKGVG-----------------------------
S. pistillata A1 51 ELKVAKFDFAYVAGPLT--IIVWILIASFAKLVFHLSHKLSSIVPESCLVIVLGIVIGGIMKGIG-----------------------------
S. pistillata A6 37 AEKVEK---YSAIDSAFLLVMMFLLIVTVLTVWL-FKVKRFRFLHETGVCMIYGMIIGFLIKYISGN---KRVAISHPNCTITSPTKKLYISLSN
S. pistillata A7 43 EDEMEN---THKTDSLSMLILITLLIINVLMIWL-FKIRRFEIFHETGVAMILGVVVGAIIKYSESRGEKKPLAVKLKNCGNITIAPKNVFVNIN
S. pistillata A8 76 PPEVEQ---HSSMTIFFILL---VVALCILLIHF-LLKTKFHYLPESVAVIFLGALVGLVIKALSHF--------------------------
* ** * S3 S4
H. sapiens A1 151 -----------------------E--TPPFLQSDVFFLFLLPPIILDAGYFLPLRQFTENLGTILIFAVVGTLWNAFFLGGLMYAVCLVGGEQI
S. pistillata A1 114 -----------------------ILEIVPTFSSRTFFLFMLPPIVLEAGYFLQDRAFFNNIGTILLYAVVGTIFNTFTVGLSLFGVSLTGGLN-
S. pistillata A6 125 GSKYSYTLLGQV---QDSSLDQGSELEQMAVFDPEIFFYVLLPPIIFYAGYDMKKRFFFRNIGAILTYAFVGTTISCIVFGGTIYAYTKFDSSVV
S. pistillata A7 134 GTEYSYTLTGIRFPPHTSPPDEGNEIESKSSFNPEIFFYVLLPPIIFYAGYSFQKRYFFRNLGAIMTYAFFGTTISCIVTGSMVFGFNIWT-GVT
S. pistillata A8 136 --------------------ALGDWRKEEHFNPTAFFLILLPPIIFESGYSLHKGNFFANIGSIIVFAIFGTAVSAIVIGGGIYLLGK--GGVA
S5 S6 S7
H. sapiens A1 220 NNIGLLDNLLFGSIISAVDPVAVLAVFEEIHINELLHILVFGESLLNDAVTVVLYHLFEEFANYEH-----VGIVDIFLGFLSFFVVALGGVLVG
S. pistillata A1 184 --LTLMHTLTFAALIAAVDPVAVLAVFEEIHVNVMLYILVFGESLLNDAVTVVLYHLFEGLAGFDE-----VSYKEILTGFASFFVVSLGGTTLG
S. pistillata A6 217 EGFDFLECLIFGSLISATDPVTVLAIFHDLHVDVDLYALVFGESVMNDAVAIVLFRSVETYLDKGS----GFQVNRLFESVGVFIGVFSGSFFLG
S. pistillata A7 228 EDFDYAECLLFGALISATDPVTVLAIFHDLHADLDLYALVFGESVLNDAVAIVLYRAIETYLAYNQSDLRDFDLFSFLKAIADFVAIFVGSFTIG
S. pistillata A8 208 FQLDLRESFAFGSLISAVDPVATLAIFHALDVDPTLNMLVFGESILNDAVSIVMTNTILEMGAAQYADS--SSFETFFSAVGNFLVMFCGSAGIG
S8 S9
H. sapiens A1 310 VVYGVIAAFTSRFTSH--IRVIEPLFVFLYSYMAYLSAELFHLSGIMALIASGVVMRPYVEANISHKSHTTIKYFLKMWSSVSETLIFIFLGVST
S. pistillata A1 272 LLWGLATAFVTKYTDH--VRVIEPIFVFIMSYLSYLTAELFHLSGIMSIVTCAIVMKPYVERNISRKSHTTIKYFLKMLSSSSETLIFMFLGVQV
S. pistillata A6 308 FGMGLVTALVTKLTKLSDFPLLETALFFLLSWTTFLMAEAANLTGIVAVLFCGISQAHYTYNNLSEESKQNTKQTFALLNFLAESFIFSYMGLSL
S. pistillata A7 323 CGMSFVNALVTKLTKIGSFPILETALFFLMSYCTFLAAELSNWSGIVAVLFCGITQQHYTYKNLTEESRMRTTQTFELLNFLAETFIFSYIGLSV
S. pistillata A8 301 ILFALISAFLLKHVDLRTTPSLELGTMLIFSYAPYGLAEGLKLSGIMAILFCGIVMSHYTHFNLSPMTQITVQQIFRTTAFMAETCVFAYLGMAI
S10 * S11 ** S12
H. sapiens A1 403 VAGS-HHWNWTFVISTLLFCLIARVLGVLGLTWFINKFR-IVKLTPKDQFIIAYGGLRGAIAFSLGYLLDKKHFPMCDLFLTAIITVIFFTVFVQ
S. pistillata A1 365 VSWS-HEWNTQFVFVTLVFLLVFRALGVVILTFLANRFGRINKLSAVDQFIMSYGGIRGAVSFSLAVLLDEHHFPMKNMFVTTIVIVLFTVFFQ
S. pistillata A6 403 FTFQHHQWNFGFISWTFLAMTLGRLLNVYPLSFLLNLGR-HRKISYKFQHMMVFAGLRGAVAFALAMRNTE-SVPRQ-MMLTSVLVVVLVTVIFN
S. pistillata A7 418 FAFTNHQWNVGFIGWSFMAIQVGRALNVYPLSLFLNLGR-TRKIPLNVQHMMFFSGLRGAIAFALAVRNTV-SIPRQ-MMLTTTLIIVIVSVIFF
S. pistillata A8 396 FSFK-HQFRPAFVIWTIILCLLGRAVNIYPLSSLVNQFR-DVRISRKTQFIMWFSGLRGAVAFALVLHLQLDDEKRH-VLITSTLIIIMFTILCL
Glycine rich region
H. sapiens A1 496 GMTIRPLVDLLAVKKKQETKRSINEEIHTQFLDHLLTGIEDICGHYGHHHWKDKLNRFNKKYVKKCLIAGERS--KEPQLIAFYHKMEMKQAIEL
S. pistillata A1 459 GMTIKPLVRILHVKLRGKEQLSMCVELNEKLLDHLVAGIEEISGHHGHGYYWEMLEYVHTHYLRRWLIRDNDRFVQDEEILLAYRRLAYKDALTR
S. pistillata A6 495 GGATMSVLSALKIRVGVDPEEEEKQ---STYVSN-----GD-----------ENLRSAQTHYERAWIFRKWYDFDVKYMKPIFTNYGQ-PLTETL
S. pistillata A7 510 GGVTMPVLSCLKIKTGIDEHEEEKK---ARRLSL-----ID---VNDPSASTPQERIEKRQYEKSWLVRVWSRFDEKYLTPIFVEESQ-IEPPSS
S. pistillata A8 488 GGSTLPLLKLLKADQGLEKSLTLSK---TQT--------------EGKAVDADQLTDDEWRISSKKALKGFSRLDAKYFIPFFTRKFT-RQEVR-
H. sapiens A1 589 VESGGMGKI--PSAVSTVSMQNIHPKSLPSERILPALSKDKEE---
S. pistillata A1 554 LEKEGSGSAIFPTNIPL---ELLAKSAFEASAICANESDEDEDENKPRLRAGEDVRVPDAIQVDWRASGVPSG--------GELEIHDTSMHSLN
S. pistillata A6 570 PGWCG-------------PLARFLSRSRPDAK--TETVATDSD--EDMLNINGQLSFTSEVMVRPASGSVNSGEPPDQMREGDLGLGAEQMTTRA
