# Supplementary material for: Intracellular pH regulation: characterization and functional investigation of H+ transporters in Stylophora pistillata
Source: BMC Mol Cell Biol. 2021 Mar 8;22:18. doi: 10.1186/s12860-021-00353-x (PMC7941709; doi:10.1186/s12860-021-00353-x)
Supplement: Supplementary file 6 — Additional file 6. Sequence comparison of the S. pistillata and H. sapiens SLC9C1 proteins. The boxes represent human voltage-sensing domains (S1-S4), and the asterisks indicate conserved positively and negatively charged residues relevant to voltage sensing. The rectangles indicate conserved positively charged residues in S4 that are present in S. pistillata but missing in hSLC9C1. The triangles indicate residues involved in the cyclic nucleotide-binding domain. [file 12860_2021_353_MOESM6_ESM.pdf]

```
S. pistillata    1 -------------------------------------------------------------------YTSMSSQNPHLILTTFLPVLLFESAFAM
H. sapiens       1 MAGIFKEFFFSTEDLPEVILTLSLISSIGAFLNRHLEDFPIPVPVILFLLGCSFEVLSFTSSQVQRYANAIQWMSPDLFFRIFTPVVFFTTAFDM

S. pistillata   29 DVHIFYKMFWQVVLLAVFGLAAATALSG----VMAKMVFVDYHWTWLEALLFGSIVSATDPVAVVALLNDLGTSKQLSTIIEGESLLNDGMAIVL
H. sapiens      96 DTYMLQKLFWQILLISIPGFLVNYILVLWHLASVNQLLLKPTQW-----LLFSAILVSSDPMLTAAAIRDLGLSRSLISLINGESLMTSVISLIT

S. pistillata  120 YKIFFNLAFSSM------TATEIGLYFPRVALGGFFFGLVAGRVTVFWLQHVFNDALVEITITLASTYLTFYIGEEVLGISGVIAVVMLGIQINA
H. sapiens     186 FTSIMDFDQRLQSKRNHTLAEEIVGGICSYIIASFLFGILSSKLIQFWMSTVFGDDVNHISLIFSILYLIFYICE-LVGMSGIFTLAIVGLLLNS

S. pistillata  209 LKTSISPEVEVFLHRFWEMLAYLANTLIFIMVGVVIMEKALNSLNEYDLFLLVVDYFGITVIRGLVMMTFSPILMRLGYGLSWQNAVVAAWGGLR
H. sapiens     280 TSFKAA-IEETLLLEFWTFLSRIAFLMVFTFFGLLIPAHTYLYIEFVDIYYSLNIYLTLIVLRFLTLLLISPVLSRVGHEFSWRWIFIMVCSEMK

S. pistillata  304 GAVGLALALQVYIDHPLIG-----GKLLAHTAGIVMFTLLVNATTMKKLLEKLGMSEISDAKKIAMANAVRQVQESNQRTLTMLKADRFLAGADW
H. sapiens     374 GMPNINMALLLAYSDLYFGSDKEKSQILFHGVLVCLITLVVNRFILPVAVTILGLRDATSTKYKSVCCTFQHFQELTKSAASALKFDKDLANADW

S. pistillata  394 DIAERDCEVHNPYVEVDDEHAKETPFLRRLSTCPNCESSVPNEPSAQEFAEMANDGRVRLIKALKVSYWKQFEHGMLSREAVQTLINLADTAMDE
H. sapiens     469 NMIEKAITLENPYMLNEEETTE-----HQKVKCPHCNKEIDEIFNTEA----MELANRRLLSAQIASYQRQYRNEILSQSAVQVLVGAAESFGEK

                                                                                                S1                S2
S. pistillata  489 EGRFIDIDDLQSYWSVPPFLQKIKDKLEQMKQTKPTEHIPPPNNKILAFMYVVAVHVMFEVIVNTLIVINMVPIVLELSSDDDAPYMETLTIINY
H. sapiens     555 KGKCMSLDTIKNYSESQKTVTFARKLLLNWVYNTRKEKEGPSKYFFFRICHTIVFTEEFEHVGYLVILMNIFPFIISWISQLNVIYHSELKHTNY

                                                            S3                      S4
S. pistillata  584 IYCSIYIAEATWKSIALAFRRFYFKDYWNLIDLVALS--IVDIVIDEVADKATGSFSPSVLKVAKVFRVLRMGRLVRLFKTLLPRLINSVNDIIN
H. sapiens     650 CFLTLYILEALLKIA--AMRKDFFSHAWNIFELAITLIGILHVILIEID---TIKYIPNETEVIVFIKVVQFFRILRIFKLIAPKLLQIIDKRMS

S. pistillata  677 RQLSFGYDVGKGYIIAEEEVIKLIDHMVVDKRIAKDLKQRSEQSRLDVVKSLGMLQREHPGIAISVKTRQAIRTILNNARDVIHELKGGGLLDEA
H. sapiens     740 HQKTFWYGILKGYVQGEADIMTIIDQITSSKQIKQMLLKQVIRNMEHAIKELGYLEYDHPEIAVTVKTKEEINVMLNMATEILKAFGLKGIISKT

S. pistillata  772 EAVKLESLVEVKMKRQLSAPTSISPQKPTELLRNVVWLEGMPSEAVEFVTSAARIKMFEAGDTIARQGEDLKGIYLIVSGMVKIIGVSVARRSCF
H. sapiens     835 EGAGINKLIMAKKKEVLDSQSIIRPLTVEEVLYHIPWLDKN-KDYINFIQEKAKVVTFDCGNDIFEEGDEPKGIYIIISGMVKLEKSKPGLGIDQ

S. pistillata  867 DGEEPEVGGMIVTTDYVSAGNLIGEMGLLTSSQRNSSCTCESAVQAYFITIDDMKVAMS-RYPDLEDRLWKVCGVRIAVPLLLEVPAYYSWTKDK
H. sapiens     929 MVESKEKDFPIIDTDYMLSGEIIGEINCLTNEPMKYSATCKTVVETCFIPKTHLYDAFEQCSPLIKQKMWLKLGLAITARKIREHLSYEDWNYNM

S. pistillata  961 IRVMCERSFIVNLSQGSNTIFKTNEQMKEVILIQGKVTDLESRDIYEGPCVLPKIYRTFRLHFDGVEPKILVIARFHETALPDDPADNPSALDLV
H. sapiens    1024 QLK-LSNIYVVDIPMSTKTD-IYDENLIYVILIHGAVEDCLLRKTYRAPFLIPITCHQIQSI-----------EDFTKVVIIQTPINMKT----F

S. pistillata 1056 GLNAERRY----RSNSDPSSSQIFGGTEATFRGKRRLSTGGRAMSVDESRHRLDPIPDRLQGRKIVVSSMVEDDKAMSSMNATANDIPKKEKHGL
H. sapiens    1102 RRNI-RKFVPKHKSYLTPGLIGSVGTLEEGIQEERNVKEDGAHS----AATARSPQPCSLLGTKFNCKE-----------------SPR------
```
