# Supplementary material for: Intracellular pH regulation: characterization and functional investigation of H+ transporters in Stylophora pistillata
Source: BMC Mol Cell Biol. 2021 Mar 8;22:18. doi: 10.1186/s12860-021-00353-x (PMC7941709; doi:10.1186/s12860-021-00353-x)
Supplement: Supplementary file 7 — Additional file 7. Number of phosphorylation sites and N-glycosylation sites predicted for S. pistillata H+ transporters. [file 12860_2021_353_MOESM7_ESM.pdf]

Protein	Post-translational modifications	
	N ° Phosphorylation sites	N-Glycosilation sites
**SLC9A1**	24	N33, N183, N332 and N586
**SLC9A6**	17	N108, N124, N349 and N370
**SLC9A7**	14	N120, N133, N292, N364 and N385
**SLC9A8**	12	N41, N48 and N147
**SLC9B1**	7	N279 and N365
**SLC9B2**	7	N293
**SLC9C**	27	N340, N972, N1132 and N1155
**V_0 V-ATPase subunit-a**	13	N286 and N378
**H_vCN 1.1**	4	N48
**H_vCN 1.2**	6	-
