# Supplementary material for: Intracellular pH regulation: characterization and functional investigation of H+ transporters in Stylophora pistillata
Source: BMC Mol Cell Biol. 2021 Mar 8;22:18. doi: 10.1186/s12860-021-00353-x (PMC7941709; doi:10.1186/s12860-021-00353-x)
Supplement: Supplementary file 8 — Additional file 8. A. V0 V-ATPase subunit-a isoforms in coral S. pistillata. B. Human and coral homologs of V-ATPase subunits. [file 12860_2021_353_MOESM8_ESM.pdf]

A.

Accession Number	aa	EST[1]	Coding Sequence[1]
XP_022795094.1	853 aa	Spi_isotig04722 gene=isogroup01952 length=3014	101-2614
XP_022795103.1	843 aa	Spi_isotig04722 gene=isogroup01952 length=3014	101-2614
XP_022793418.1	761 aa	Spi_isotig04722 gene=isogroup01952 length=3014	380-2614
XP_022793433.1	747 aa	Spi_isotig04722 gene=isogroup01952 length=3014	422-2614

B.

Subunit	*H. sapiens*[1,2]	*S. pistillata*	
**V_1 domain**		**Protein**	**Scaffold**
**A**	NP_001681.2	XP_022805266.1[2]	561
**B**	NP_001684.2; NP_001683.2	XP_022790505.1[2]	144
**C**	NP_001686.1; NP_001034451.1	PFX13914.1[2]	871
**D**	NP_057078.1	Spis21337[3]	771
**E**	NP_001687.1; Q96A05.1	Spis7193[3]	90
**F**	NP_004222.2; NP_001185838.1	Spis12239[3]	214
**G**	NP_004879.1; NP_569730.1; NP_573569.1	PFX21706.1[2]	266
**H**	NP_057025.2	PFX17032.1[2]	502
**V_0 domain**			
**a**	NP_001123492.1; NP_036595.2; NP_006010.2; NP_065683.2	XP_022795094.1[2]	8
**d**	NP_004682.2; NP_689778.1	Spis14606[3]	298
**e**	NP_003936.1; NP_001276919.1	Spis13055[3]	240
**c**	NP_001185498.1	SpisGene5214[3]	57
**c''**	NP_001281262.1	Spis4394[3]	45
**Ac45**	Q15904.2	Spis842[3]	5

[1]Toei et al., 2010; [2]GenBank; [3]Bhattacharya et al., 2016
