# Supplementary material for: Intracellular pH regulation: characterization and functional investigation of H+ transporters in Stylophora pistillata
Source: BMC Mol Cell Biol. 2021 Mar 8;22:18. doi: 10.1186/s12860-021-00353-x (PMC7941709; doi:10.1186/s12860-021-00353-x)
Supplement: Supplementary file 9 — Additional file 9. Statistical analysis of the relative mRNA quantification (Rq) of SLC9s, V0 V-ATPase subunit-a and HvCNs (N = 3) in S. pistillata total (total colony) and oral (oral fraction) fractions. [file 12860_2021_353_MOESM9_ESM.pdf]

Gene	Oral		Total		p-value
	Mean	Std Dev	Mean	Std Dev	
**SLC9A1**	1.13	0.308	0.908	0.0736	0.289
**SLC9A6**	1.49	0.3704	0.691	0.117	0.0235*
**SLC9A7**	1.84	0.575	0.563	0.05101	0.0183*
**SLC9A8**	0.861	0.145	1.19	0.235	0.108•
**SLC9B1**	0.942	0.265	1.11	0.291	0.487
**SLC9B2**	1.0903	0.321	0.955	0.126	0.535
**V_0 V-ATPase subunit-a**	1.066	0.161	0.945	0.08000208	0.31
**H_vCN 1.1**	0.867	0.122	1.176	0.227	0.107•
**H_vCN 1.2**	1.23	0.135	0.818	0.043	0.00735**
