# Supplementary material for: Intracellular pH regulation: characterization and functional investigation of H+ transporters in Stylophora pistillata
Source: BMC Mol Cell Biol. 2021 Mar 8;22:18. doi: 10.1186/s12860-021-00353-x (PMC7941709; doi:10.1186/s12860-021-00353-x)
Supplement: Supplementary file 10 — Additional file 10. Statistical analysis of the relative mRNA quantification (Rq) of SLC9s, V0 V-ATPase subunit-a and HvCNs (n = 5) at pH 8.1 and 7.2 after 1 week and 1 year of pCO2 exposure. The asterisks indicate significant differences (** p < 0.05). [file 12860_2021_353_MOESM10_ESM.pdf]

Gene	pH 8.1				pH 7.2				p-value 1 week	p-value 1 year
	Mean		Std Dev		Mean		Std Dev			
	1 week	1 year	1 week	1 year	1 week	1 year	1 week	1 year		
**SLC9A1**	1.12	0.902	0.0575	0.0989	0.987	1.12	0.0601	0.158	0.1572	0.0296*
**SLC9A6**	1.0474	0.974	0.0942	0.259	0.966	1.05	0.0474	0.0932	0.123	0.521
**SLC9A7**	1.0002	0.988	0.134	0.234	1.04	1.05	0.319	0.256	0.779	0.657
**SLC9A8**	0.975	0.997	0.112	0.288	1.029	1.03	0.146	0.118	0.527	0.779
**SLC9B1**	0.981	0.984	0.182	0.405	1.0058	1.10	0.127	0.233	0.814	0.568
**SLC9B2**	1.041	0.916	0.107	0.226	0.959	1.15	0.0486	0.314	0.158	0.202
**V_0 V-ATPase subunit-a**	1.066	0.981	0.1103	0.117	0.932	1.03	0.0787	0.114	0.575	0.521
**H_vCN 1.1**	1.16	1.11	0.324	0.365	0.9208	0.949	0.0596	0.1804	0.138	0.388
**H_vCN 1.2**	1.0014	1.014	0.165	0.317	1.009	1.03	0.0696	0.168	0.927	0.902
