# Supplementary material for: Intracellular pH regulation: characterization and functional investigation of H+ transporters in Stylophora pistillata
Source: BMC Mol Cell Biol. 2021 Mar 8;22:18. doi: 10.1186/s12860-021-00353-x (PMC7941709; doi:10.1186/s12860-021-00353-x)
Supplement: Supplementary file 11 — Additional file 11. List of S. pistillata H+ transporter genes with RT-PCR primers and product sizes. [file 12860_2021_353_MOESM11_ESM.pdf]

Target gene	Reference	Real-time PCR primers		Product size - base pairs (bp)
SLC9A1	*Capasso et al.*	F	TCATTCTCCTTGGCTGTTTTACTG	138 bp
		R	CATGAAGAATCCGCACAAGAGGTT	
SLC9A6		F	GTGGACATGGGGCCGCTTCTAT	132 bp
		R	AATCTGCAGCCGCTTGAATCTCG	
SLC9A7		F	CGCCGGACGAAGGAAACGAA	140 bp
		R	AATGGCGCCTAAATTACGAAAGAAA	
SLC9A8		F	AGCCGTGGTCGTTCTCTTAGTCGT	116 bp
		R	CATCCCCCTTGGTGTTGTTCGT	
SLC9B1		F	CCGCGTTTGGTGGCTTTTTA	113 bp
		R	CGCAACATTGATCCCTGGCAC	
SLC9B2		F	CTTGATTTGGGCGGTGTTATGGTC	141 bp
		R	GGAGGTAGATGGAGGTAGGGGATTT	
SLC9C		F	CAAAGGTGGTGGGTTGTTAGATGAG	106 bp
		R	TTGTGGGGAAATGGAGGTTGG	
V_0 V-ATPase subunit-a		F	GAGGAGTTTGATTTTGGGGAAGTA	97 bp
		R	AGTCGCAAATAAGAGGCTGTGTT	
H_vCN1.1		F	CAGGATGATCAGCCACTTGTTG	137 bp
		R	AGGCCTTTCGTCTTCATTTTCT	
H_vCN1.2		F	CAAGTTTTACGCGATGGGATGC	121 bp
		R	CCTTCTTTGCGCGGATAACG	
36B4	*Moya et al., 2008*	F	AACAAGGTGGCAGCCCCAGC	91 bp
		R	GTCTTCTCGGGACCCAGGCCA	
L40	*Zoccola et al., 2016*	F	CGACTGAGGGGAGGAGCCAA	94 bp
		R	CTCATTTGGACACTCCCTT	
