# Supplementary material for: Intracellular pH regulation: characterization and functional investigation of H+ transporters in Stylophora pistillata
Source: BMC Mol Cell Biol. 2021 Mar 8;22:18. doi: 10.1186/s12860-021-00353-x (PMC7941709; doi:10.1186/s12860-021-00353-x)
Supplement: Supplementary file 5 — Additional file 5. Sequence comparison of the S. pistillata and H. sapiens SLC9B proteins. [file 12860_2021_353_MOESM5_ESM.pdf]

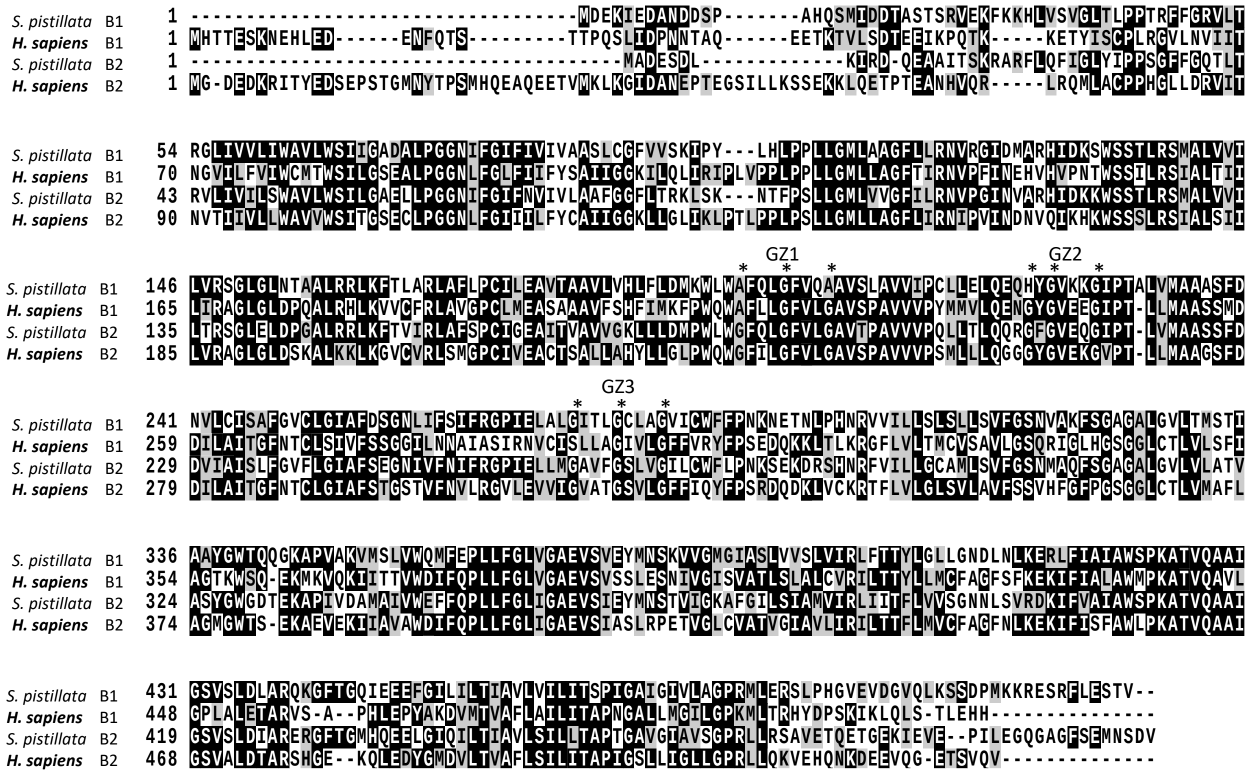

S. pistillata B1
H. sapiens B1
S. pistillata B2
H. sapiens B2
GZ1
GZ2
GZ3
